# Early phases of COVID-19 management in a low-income country: Bangladesh

**DOI:** 10.1017/ice.2020.147

**Published:** 2020-04-17

**Authors:** Mohammad R. Monjur, Md. Zakiul Hassan

**Affiliations:** 1 University of Newcastle, New South Wales, Australia; 2 International Centre for Diarrhoeal Disease Research, Dhaka, Bangladesh

*To the Editor—*The World Health Organization has emphasized the importance of diagnostic testing in tracking and managing COVID-19, and most high-income economies have adopted widespread population testing schemes. The United States now leads the way, with >370,000 tests performed as of March 26, 2020.^1^ This level of testing starkly contrasts with low-income economies such as Bangladesh, where an almost contrarian strategy seems to have been adopted that is arguably masking the true national spread of the virus.

From the first reported case of COVID-19 in Bangladesh on March 8 until March 28, 1,068 samples were tested by the Institute of Epidemiology, Disease Control and Research (IEDCR) in Dhaka.^2^ The IEDCR was the sole institute in Bangladesh with testing facilities for COVID-19 until March 26, when a second facility was given testing rights. Centralized testing in these underresourced public institutions has been unable to effectively respond to the wave of suspected COVID-19 patients. Even at this initial stage with limited confirmed cases, busy telephone hotlines and lack of timely testing for symptomatic patients raised concerns regarding Bangladesh’s preparedness. In addition, the Bangladesh government has not sought to proactively limit community transmission from primary cases thus far. With a population of 161 million and a total of 1,169 ICU beds,^3^ this inadequate strategy could potentially devastate Bangladesh’s health system with multiple outbreaks.

This risk is compounded by thousands of Bangladeshi workers returning from COVID-19–struck countries and poor adherence to self-quarantine recommendations due to limited education and monitoring mechanisms. This situation is particularly problematic for Bangladesh because a significant portion of returning workers (ie, significant sources of SARS-CoV-2) reside in rural areas outside Dhaka and thus carry the virus to some of the most vulnerable and ill-equipped communities. This situation was likely worsened by the government declaring a 10-day holiday without travel restrictions from March 26 to April 5, which encouraged millions of city workers to leave Dhaka and return to their rural communities.^4^

We believe that Bangladesh has lacked coordinated policy decision and enforcement measures to curtail COVID-19 transmission thus far. We urge policy makers to follow WHO guidance and observe other countries’ experiences, which point to a strategy of acting decisively, quickly, and early, well before case numbers reach a crisis level for containment. We believe Bangladesh has not yet reached this point, so urgent implementation of a coordinated policy may prevent a spike in cases that is likely to stretch Bangladesh’s health system well beyond its capacity.
